# Addressing historic environmental exposures along the Alaska Highway

**DOI:** 10.3402/ijch.v72i0.21187

**Published:** 2013-08-05

**Authors:** Anna Godduhn, The Northway Health Study Team, Lawrence Duffy

**Affiliations:** 1 Department of Chemistry and Biochemistry, University of Alaska Fairbanks, Fairbanks, AK, USA; 2 Cheri Marunde, Polly Hyslop, Jane Fix, Howard Fix, and Becky Gallen: Northway, AK, USA

**Keywords:** endocrine disruption, persistent organic pollutants, military waste, environmental toxics, complexity, uncertainty

## Abstract

**Background:**

A World War II defense site at Northway, Alaska, was remediated in the 1990s, leaving complex questions regarding historic exposures to toxic waste. This article describes the context, methods, limitations and findings of the Northway Wild Food and Health Project (NWFHP).

**Objective:**

The NWFHP comprised 2 pilot studies: the Northway Wild Food Study (NWFS), which investigated contaminants in locally prioritized traditional foods over time, and the Northway Health Study (NHS), which investigated locally suspected links between resource uses and health problems.

**Design:**

This research employed mixed methods. The NWFS reviewed remedial documents and existing data. The NHS collected household information regarding resource uses and health conditions by questionnaire and interview. NHS data represent general (yes or no) personal knowledge that was often second hand. Retrospective cohort comparisons were made of the reported prevalence of 7 general health problems between groups based on their reported (yes or no) consumption of particular resources, for 3 data sets (existing, historic and combined) with a two-tailed Fisher's Exact Test in SAS (n=325 individuals in 83 households, 24 of which no longer exist).

**Results:**

The NWFS identified historic pathways of exposure to petroleum, pesticides, herbicides, chlorinated byproducts of disinfection and lead from resources that were consumed more frequently decades ago and are not retrospectively quantifiable. The NHS found complex patterns of association between reported resource uses and cancer and thyroid-, reproductive-, metabolic- and cardiac problems.

**Conclusion:**

Lack of detail regarding medical conditions, undocumented histories of exposure, time lapsed since the release of pollution and changes to health and health care over the same period make this exploratory research. Rather than demonstrate causation, these results document the legitimacy of local suspicions and warrant additional investigation. This article presents our findings, with discussion of limitations related to study design and limitations that are inherent to such research.

In the late 1800s, people of the Upper Tanana River region were well adapted to the extreme conditions of Subarctic wetlands (1). Whitefish, small and large game, and plants from root to berry provided high quality nutrition. Early in the 20th century, “white foods” such as sugar, flour and tea were brought by foot from Dawson, YT Canada, some 150 km away. In the 1920s and 1930s, American cultural influence increased – bringing church, school, merchants and the fur trade. Epidemics brought heavy losses of life. Despite the changing context, food was acquired almost entirely from the land (1, 2).

Northway [Fig. 1 (3–5)] was a large military base during World War II (6, 7). Opened in 1942 and paved in 1944, the Northway airfield fueled some 8,000 flights along the Alaska–Siberia Route (ALSIB). Northway also served as a staging area for the 1942 construction of the Alaska–Canada Route (ALCAN), a road built to supply the air route. Local residents generally supported the defense strategy, including the international road that would provide better access to extended family and supplies (8). Food staples, alcohol, tobacco, fuel and petrochemicals became common place (9).

**Fig. 1:**
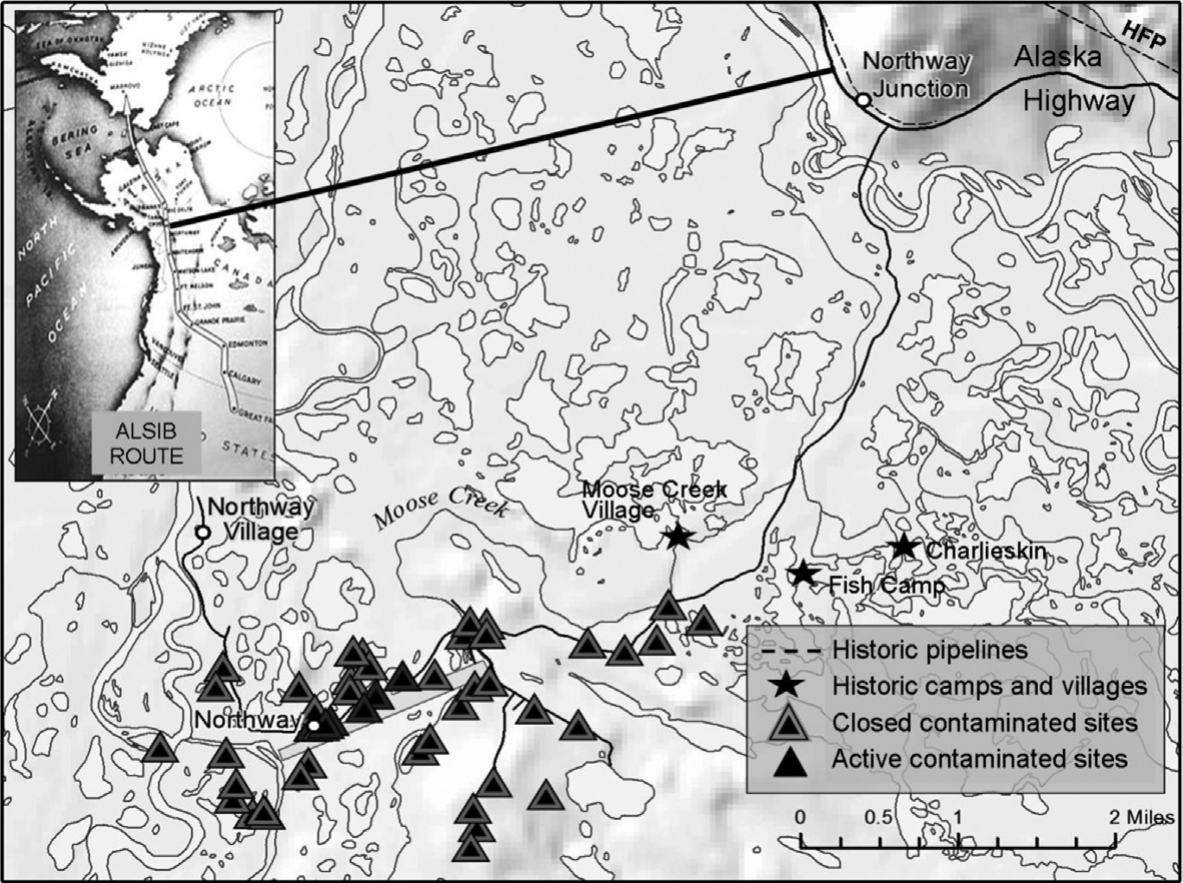
Northway Village, Northway and Northway Junction; not all pipelines or contaminated sites are shown (3–5). The western portion of the airfield drains to the north, while the east end drains to Moose Creek. An asphalt plant near the east end of the airfield was demolished in the 1950s, but open tar mounds remained on the banks of Moose Creek until the 1980s. Tens of thousands of yards of soil and thousands of tons of equipment and debris were removed in the 1980s and 1990s; soil was burned and redistributed. Most sites have been closed to further remedial action; some are still open, especially for monitoring petroleum plumes.

The Army left the area in 1945 and the ALCAN became the Alaska Highway. The Federal Aviation Administration (FAA) station remains open, with decreased activity since the end of the Cold War (10). Pesticide and herbicide application along the highway and Northway Road continued for decades (11); oil pipelines that paralleled the roads often leaked and were sprayed with defoliants for access (12, 13).

Residents petitioned for inclusion on the National Priorities List and welcomed “Superfund” clean-up under the US Comprehensive Environmental Response, Compensation, and Liability Act in the 1990s. Contaminants in locally consumed wild foods were not thoroughly evaluated, and ongoing questions regarding harm to human health were never investigated (14, 15). The Northway Wild Food and Health Project (NWFHP; 16)demonstrates complexity and uncertainty, and also the potential for collective local knowledge to provide a basis for research.

## Objective

To address local questions regarding the legacy of military waste, this project included 2 distinct yet related studies. The Northway Wild Food Study (NWFS) reviewed historical data and identified likely pathways of exposure to toxics over time. The Northway Health Study (NHS) provided the first investigation of locally suspected links between historic resources and ongoing health problems, using available data consisting of personal knowledge related to historic resource uses and health problems.

## Design

Following consultation with Gerald Albert, the then-President of the Northway Village Council, and UAF-IRB approval (UAF 05-31 and 09-06), the lead author conducted a set of 36 open-ended interviews with 44 residents in 2005 and 2006. Regular users of wild resources were guarded, but willing to describe their concerns. Those initial respondents ranked species for contaminants testing and called for an investigation of health. In 2009, 2 additional surveys collected data for the retrospective cohort health study: a self-administered questionnaire and another set of interviews, described below.

Although health was not queried during the initial interviews, cancer was consistently identified as the primary concern related to contaminants and was the only health problem clearly linked by respondents to a particular resource: Moose Creek (1940–1980s). Thyroid problems, learning disabilities, autoimmune diseases and reproductive problems were suspected of being related. Whitefish (1940–present), the Haines Fairbanks Pipeline (HFP; 1970s–2000), and water from the tank at the Federal Aviation Administration facility (1950s–1980s) were suspected contributors. Vague concerns were also raised about berries and edible roots gathered along the road, and muskrat harvested north of the airstrip until the 1970s.

Described health problems echoed those found in the toxicological literature (17, 18), and remedial documentation and limited existing contaminant levels data (19–22) collected by the US Fish and Wildlife Service (USFWS) gave credence to concern. It was emphasized that a broad study based on local knowledge might not have the statistical power to “find” relationships between resource uses and illness; if relationships were found, uncertainty would preclude definitive conclusions.

The volunteer Health Study Team (HST) decided to conduct a retrospective cohort study that would compare the reported prevalence of general health problems between groups based on their reported consumption of particular resources. Participation rates would likely have been impaired by requirement of medical records, so personal knowledge was used as data. Three data sets resulted from 2 collaboratively designed surveys: Existing Households included people living in Northway (2009); Historic Households included people who had moved or passed away (1940–2008); and Combined Households (duplicates removed) for more robust statistics.

## NHS data collection

### Existing households

In the spring of 2009, a self-administered questionnaire was sent to all 90 households in the Northway area (identified by the Northway Postmaster, a member of the HST) with a newsletter describing the project. The questionnaire collected information about demographics, food, and water resources used over time (Table I), and the health of household members. General health problems queried among existing households included (a) cancer of any type or site, (b) thyroid problems (hypo-, hyper- and undefined), (c) reproductive problems (unsuccessful pregnancies and birth defects), and (d) diabetes.

**Table I:** Resources evaluated for association with health disorders

Resource	Users: non-users, combined data	Primary known concerns (possible)	Time range of use
MC water^a^	116:140	Petroleum, polychlorinated biphenyls, pesticides	Pre-war–1980s
FAA water^b^	126:127	Chlorinated byproducts of disinfection	1960s–1980s
Fish^c^	240:75	Polychlorinated biphenyls, pesticides, mercury	pre-war–present (less now)
Beef	163:39	Saturated fat (hormones, antibiotics)	1970s–present (more now)
Caribou^d^	86:149	Radioactive fallout from atmospheric nuclear testing	1950s–1970s
Roadside Rts^e^	174:85	Lead [Pb]	1950s–present (less now)
HFP^f^	139:96	Herbicides including 2,4,5-T	1970s–2000

a Moose Creek (MC; *Dzį*įł*ay Niign*) was redirected and heavily impacted during WWII. MC moves east from the airstrip and former asphalt plant and tar mounds, roughly parallel to Northway Road (Fig. 1).

b Federal Aviation Administration personnel often allowed public-use of the facility's water well and holding tank.

c Broad and Humpback Whitefish (Ł*uugn;* Coregonus nasus, and C. oidschian) are a traditional source of protein and dietary oil, and a symbol of cultural identity in the Upper Tanana.

d Caribou (*Utsi; Rangifer tarandus*) in the era of atmospheric nuclear weapons’ testing was a possible concern. Caribou was the only compared resource not positively associated with any problem and showed marginal negative association with high BP and high cholesterol. (There was no comparison group for moose users.)

e Edible roots or “Indian Potato” (*Tsüü; Hydysarum alpinum*) grow well in coarse and compact roadside soils.

f The Haines Fairbanks Pipeline (HFP) corridor was used for traveling and collecting many resources. The raspberries were so good people camped in the corridor and also harvested basket roots, edible roots, mushrooms, firewood and sometimes moose.

“Household” was defined as a group of people living together. Study team members participated as informants, solicited neighbors and assisted some households with the questionnaire (following completion of a UAF-IRB training course). Follow-up phone calls from the lead author focused on (a) clarification of resource uses, especially historic caribou, (b) use of the Haines-Fairbanks Pipeline corridor for collecting resources and (c) a possible “historic household interview.” There were direct and open-ended questions in both the questionnaire and interview.

### Historic households

The Diabetes category grew to include less severe Blood Glucose disorders, and 3 other conditions were added to historic household interviews because they had been reported in existing households: (a) cardiac irregularities (murmurs, arrhythmias, long Q-T, and undefined irregular heartbeats), (b) high blood pressure (HBP), and (c) high (low density lipoprotein) cholesterol. The lead author interviewed 24 individuals who had knowledge of a household that no longer existed. These informants were mostly older adults who described their own young-adult household and sometimes their childhood household. Uncertain information (e.g. “I think dad had …”) was excluded if not confirmed by another family member.

## Data preparation, combination and analysis

Many realities preclude reliable estimates of historic resource consumption, so responses were not scaled for age-at nor extent-of use and were analyzed as binary (yes or no) data. Household members were assumed to have used the same resources as the informant during the time they lived there, unless otherwise indicated. If information on the use of a resource was not recorded, or an individual was not in the household when the resource was consumed, they were not included in the comparison (e.g. moved away before the HFP corridor was used by local residents). Information was collected for pairs of decades (e.g. 1950s and 1960s) and users were compiled into groups that had “ever” used the resource.

With 2 exceptions, sources of protein were ranked as yes for consumption if they were named by the respondent as 1 of the top 3 consumed. The first exception, whitefish, was named as the fourth protein source by 2 families. Their substantial use of whitefish was confirmed and, though they ate less whitefish in winter than most participating families, the women and children had spent much of their summers at fish camp and were ranked as fish eaters. Each family reported a case of thyroid problems.

The second exception, caribou, may have been subject to radioactive fallout in the 1950s–70s. Historic caribou was one of three resources specifically queried, and their use was independently ranked by participants (never, rare, sometimes or often). Migration changes beginning in the mid-1930s made caribou inaccessible in the Northway area. By the 1950s, some families were driving the new road to access a regional herd. Current caribou consumers were ranked as a no response if caribou was not eaten until the 1990s, when another migration shift made caribou locally accessible. Edible roots collected along the road and time spent in the HFP corridor were also independently ranked. Rare use (up to once or twice a year) was ranked as a no response.

Reports of the generalized health problem categories were not scaled by severity or timing but were likewise ranked as binary data (yes or no). Health conditions were not confirmed with medical records. Some problems, such as “his heart beat funny when he was little,” were not formally diagnosed.

Fifty individuals were present in both original datasets, and removed from the existing households’ data prior to combination. Nine individuals in responding current households were not included in comparisons because they had arrived in Northway in the past 2 years or were adopted. One infant in a historic household was not included in the comparisons because he reportedly died with an enlarged heart at 10 weeks; his death was ranked as a reproductive problem.

We compared health problems between groups of people depending on their current or former use of particular resources by determining an odds ratio (prevalence among users/prevalence among non-users). That result was statistically evaluated for likelihood, independent of other factors, with SAS statistical software (SAS 9.2 for Windows; 23).The probability of the odds ratio for the 49 comparisons in each of the 3 data sets was tested with a two-tailed Fisher's Exact test, which is designed specifically for small data sets with uneven cohorts and single digit cells (24).

## Limitations of analysis

The many historic and contemporary environmental and social factors that influence public health are not well documented in Northway, nor readily controlled. The NHS was an observational survey of health that provided a preliminary test of the general assumption of (historic) wild food safety. The study was based on 2 very general alternative hypotheses that toxic chemicals had (a) polluted local food and water sources and (b) subsequently made people sick. Suspicions were vague and there were few expectations. The data represent personal events that occurred up to 70 years ago, and include second-hand and subjective information. It is likely or at least possible that biases and mistakes exist.

While limiting the capacity for complex analyses, the reduction of information to yes or no data decreased the likelihood of scaling error – and made the study possible because retrospective measurement is unobtainable. The subjective line between rare versus some use, although defined, was an important potential source of systemic measurement error, along with response bias. Binary data regarding broad categories of illness leave the study epidemiologically weak. However, this generalization allowed information regarding spectra of illness (25) and generated more statistically useful numbers. For example, additional research including the genetic and behavioral components of cancer etiology is needed to test the implication that petroleum exposure contributed to overall cancer risk among people who used Moose Creek water.

Given likely response bias, that is, higher participation by families with disease, neither extrapolation of actual disease prevalence nor comparisons to broader populations can be made from the datasets. Other known health factors, such as stress, alcohol, tobacco, pharmaceutical prescriptions, illicit drugs, heavily processed foods and genetic vulnerabilities were ignored in the independent comparisons, but were addressed in the newsletters explaining the uncertainty surrounding the findings. For example, metabolic function is impaired by the chronic consumption of foods high in sugar and saturated fat (26, 27). This and the importance of whole foods were emphasized in our outreach.

Certainty in environmental toxicology is deeply hindered by the lack of control over highly variable human lives (28, 29). Access to alcohol, tobacco and increasingly processed food has been an important part of Northway's socioeconomic change and substantial harm is not doubted. The quality of medical care has also changed dramatically, influencing rates of diagnoses and the treatment of disease. Very limited data on fish contaminants collected by the USFWS provide the only hard, and still indirect, evidence that human exposures occurred. Pollution levels and consumption of 1950s era whitefish, for example, cannot be measured. Costly biochemical details such as levels of contaminants or biomarkers of exposure in human blood today would allow for a relative estimation of historic exposure. However, even supported by medical records, significant findings would be circumstantial.

## Results

A year 2000 study investigated organochlorines (OCs) in burbot liver (21) (whitefish is a dietary staple for burbot (*Lota lota*)). The analyses detected 10/15 pesticides tested and the most persistent of 3 Arochlor PCB blends in at least 1 of 9 samples. Several OCs were found in most samples, but only DDE, a highly persistent breakdown product of the pesticide DDT, was found in all of the burbot livers. In a more recent study, wood frogs (*Rana sylvatica*) along Northway Road were found to have rates of deformities “… among the highest reported in the published literature (22).” Frogs are not eaten by Northway residents, but both studies indicate local sources of environmental pollution (30). A thorough contaminants study of whitefish from multiple locations is a priority for Northway residents, but adequate funding was not acquired.

The return of 65% of questionnaires over 3 months (59/90 households) provided information on 178 of an estimated 223 current residents (total for 3 Census Designated Places, 31–33). Historic household interviews collected data for 197 individuals from 24 households that existed between 1940 and 2005. The number of households that existed in Northway in that period is unknown. The combined dataset totaled 325 people. Historic contaminant pathways identified by the food study were documented in hindsight, if at all. Medical details such as age-at and year-of diagnosis were not usually provided. However, with a provisional assumption that accurate data were obtained, our primary findings can be summarized as follows:

- Use of Moose Creek, which was polluted with petroleum during and following the paving of the airfield in 1944 (19), as a drinking water source (1940–1980s) was associated with cancer (OR=(24/116)/(11/140)=2.63, p=0.0026).
- Consumption of whitefish, which were polluted with persistent organic pollutants such as pesticides (20), as a major source of protein was associated with thyroid problems (OR=(13/240)/(0/75)=*i*, p=0.027) and cancer (OR=(31/240)/(4/75)=2.42, p=0.047).
- Use of the HFP corridor, where the dioxin-contaminated ingredient of Agent Orange 2,4,5-T had been sprayed (34), for collecting various resources was associated with reproductive problems and cardiac irregularities [OR=(9/139)/(0/96)=*i*, p=0.0079 and (15/139)/(4/96)=2.59, 0.053] and with less statistical strength, high blood pressure, high cholesterol and blood glucose disorders;
- Use of water from the FAA facility, reportedly heavily chlorinated, was very strongly associated with reproductive problems (OR=(16/126)/(4/127)=4.03, p=0.0042) and at least weakly associated (p<0.21) with all studied conditions.
- Consumption of edible roots collected along roadsides, where leaded fuel exhaust was released until the 1980s (35), was associated with cancer with marginal statistical strength (OR=(22/174)/(8/125)=1.97, p=0.055).

## Moose Creek and cancer

As expected by informants, the reported use of Moose Creek (1940–1980) as a water source was very strongly associated with reported all-cancer risk (OR (24/116)/(11/140)=2.63, p=0.0026). The reported users of 3 other resources also reported a higher prevalence of cancer in the combined data with decreasing statistical certainty: Whitefish [OR=(31/240)**/**(4/75)=2.42, p=0.047], FAA water [OR=(19/126)/(10/127)=1.92, p=0.054], and edible roots from along the road [OR=(22/174)/(8/125)=1.97, p=0.055].

## Whitefish and thyroid problems

All thyroid problems were reported in fish-eating families. Fish consumers showed a consistently increased risk for thyroid problems and the comparison was significant in the historical and combined datasets (Table II). Thyroid problems were associated with pipeline use (HFP) in existing households, but that comparison includes only 5 of 13 reported thyroid cases. Seven reported thyroid cases used the HFP but all consumed whitefish. Associations of fish with blood pressure and cholesterol were negative: consumers reported lower risk that was often statistically significant. These relationships were strongest in historic households [OR=(2/176)/(4/20)=0.060, p=0.0011 for high cholesterol; OR=(20/176)/(6/20)=0.40, p=0.032 for high blood pressure].

**Table II:** Odds Ratio (prevalence among users/prevalence among non-users) and p-value (likelihood of false positive) for associations between whitefish and thyroid disorders

Historic data (n=197)		Current data (n=178)		Combined data (n=325)	
		
Odds ratio	p	Odds ratio	p	Odds ratio	p
11/176 0/20	0.29	7/108 0/61	0.041	13/240 0/75	0.027

## The Haines-Fairbanks pipeline corridor and multiple health problems

Among participating historic household members, reported pipeline users reported a higher prevalence of 5 generalized problem categories. This association was not always statistically significant. In existing households, the odds ratio, or “risk,” for each of these conditions was greater than 1.5 among reported users of the pipeline, with some 80% certainty of association (p values ≤0.21, data not shown). When the 2 datasets were combined (Table III), statistics for cardiac irregularities and reproductive problems were strengthened, while statistics for the metabolic problems (high blood pressure, high LDL cholesterol and blood glucose) were weakened.

**Table III:** Odds ratio and p-value for HFP comparisons, 5 conditions, combined data (# cases)

Cardiac irregularities (9)	Repro prob (20)	High BP (29)	High cho’ (8)	Blood glucose (13)
9/139 0/96	15/139 4/96	21/139 6/96	7/139 1/96	8/139 3/96
0.0079	0.053	0.027	0.094	0.27

Other resources were associated with these health problems. In the combined dataset, FAA water users (n=126/253) had a higher risk of reproductive problems (OR=4.0, p=0.0042), cardiac irregularities (OR=3.5, p=0.084) and high cholesterol (OR=6.0, p=0.060). In historic households, people reporting store-bought beef consumption in the 1970s and 1980s (n=59/125) showed a higher risk for high blood pressure (OR=3.5, p=0.0018) and high cholesterol (OR=5.6, p=0.080), while fish eaters showed a lower risk for those problems (above). These known nutritional benefits (36) and risks (37) lend support to the data and were sustained in the combined dataset with less statistical significance (data not shown). Fish eaters also showed a higher risk of blood glucose problems that was statistically significant only among fish eaters in the 1990s and 2000s, found most strongly in the combined dataset [(8/68)/(5/134)=3.15; p=0.032].

## FAA water and multiple health problems

“Reproductive problem” was the only condition strongly associated with FAA water use, but the users reported at least a slightly higher prevalence than non-users of all the studied conditions (Table IV). While we offer no tangible evidence regarding the condition of FAA water, the unofficial public water source was often described as suspect and was likely more heavily chlorinated than today's standards allow.

**Table IV:** Odds ratio and p-value for FAA water comparisons, all conditions, combined data (# cases)

Cancer (35)	Thyroid problem (13)	Blood glucose (13)	Cardiac irregularities (9)	Reproductive problem (20)	High cholesterol (8)	High blood pres. (29)
19/126 10/127	8/126 3/127	8/126 4/127	7/126 2/127	16/126 4/127	6/126 1/127	16/126 11/127
0.054	0.11	0.13	0.084	0.0042	0.060	0.20

## Cumulative risk

**Fig. 2:**
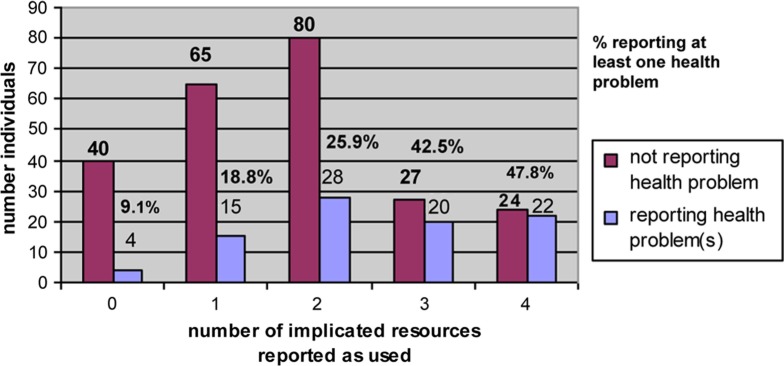
Risk of reporting at least one health problem with number of implicated resources reported. Implicated resources included Moose Creek water, whitefish, the Haines Fairbanks Pipeline corridor and FAA water.

Because the users of one resource often used others, cross-correlation between cohorts presents a potential confounder. Importantly, people who reported using more implicated resources reported a steadily increasing likelihood of at least 1 health problem (Fig. 2). This, and the observation that families who used more of those resources often reported multiple problems across generations, implies cumulative risk and may help explain the vagary of pre-study suspicions. This in no way denies the highly variable contribution of tobacco and alcohol to that cumulative risk.

## Discussion

The impossibility of measuring historic exposure levels does not negate the history. This study was a preliminary exploration of a critical component of retrospective evidence: the relative health of the people who consumed contaminated resources. The authors acknowledge epidemiological weaknesses: the study design was general because it was based on vague perceptions; cohorts were small and sometimes uneven; the data represent personal information and subjective knowledge, some of which was second hand and none of which was verified beyond other family members; and lifestyle, occupational and genetic factors were ignored in analyses.

On the other hand, it can be argued that generality of the data strengthens their credibility. The study itself was based on memories of particular events that were important to residents’ lives: drying fish, digging roots, getting water and caring for family. Measurement in retrospect was not feasible – but use, or not, of the resources is well remembered. Even with supportive direct and quantified evidence such as historic exposure levels and toxicological biomarkers, definitive conclusions would likely be impossible to draw.

Three essential facts undermine the standard components of risk assessment (dose–response, exposure assessment, risk characterization), particularly for long-term environmental exposure: (a) the variability of dynamic exposure to multiple contaminants during development is essentially infinite and begins before conception (38, 39); (b) variations in dose, especially during development, generally change the character of effects, not necessarily the likelihood or magnitude (40); and (c) variations in the exact-timing of the same dose during development also change the character of effects, rather than the likelihood or magnitude (41). Endocrine function often operates at parts per trillion concentrations, and interference during development is known to result in a constellation of problems for individuals later in life (42, 43). This is not to say that endocrine disruption is the only mechanism by which health is harmed.

The compiled observational knowledge of local residents holds substantial power to reveal patterns and trends within otherwise undocumented situations (44, 45). Patterns elucidated by collective human observations are generally found to be descriptively consistent with reality (46) and communities have a right to the insight that local application of scientific principles can provide – despite and including the uncertainty (47–49). Northway residents, well aware of the harm of alcohol and tobacco, are neither surprised by nor doubtful of the qualitative implications of this study, which will be explored in forthcoming articles.

While the findings are not definitive, the feasibility of connections between the historic pollution pathways and health problems described here support long-standing suspicions. We suggest that negligence of concerns relating environmental pollution to illness perpetuates confusion over the contemporary status of wild foods in general, within and beyond Northway. People in Northway have been left to adapt as best they can. For some that includes eliminating local fish from their diet, for others it means avoiding processed foods; some do both.

## Conclusion

There will always be uncertainty about the situation in Northway. Historic pathways of exposure to petroleum, pesticides, herbicides, chlorinated byproducts of disinfection and lead from resources that were consumed more frequently decades ago are not retrospectively quantifiable. The NHS found complex patterns of association between reported resource uses and cancer and thyroid-, reproductive-, metabolic-, and cardiac problems. The effects of environmental stimuli, including exposure to multiple contaminants via various pathways during different life stages, on genetic expression are the subject of the new field of epigenetics. New clues into the way that nature interacts with nurture challenge environmental and medical science with endless questions and elusive answers. However, there may be potential for innovative multigenerational methods of research that empower Northway residents with improved understanding and control over individual, public, and environmental health, while informing toxicological knowledge and environmental health policy more broadly.
